# Mechanical properties of femoral trabecular bone in dogs

**DOI:** 10.1186/1475-925X-4-17

**Published:** 2005-03-17

**Authors:** Thomas Pressel, Anas Bouguecha, Ute Vogt, Andrea Meyer-Lindenberg, Bernd-Arno Behrens, Ingo Nolte, Henning Windhagen

**Affiliations:** 1 Department of Orthopaedic Surgery, Hannover Medical School, Anna-von-Borries-Str. 1-7, 30625 Hannover, Germany; 2 Institute of Metal Forming and Metal Forming Machine Tools, University of Hannover, Schönebecker Allee 2, 30823 Garbsen, Germany; 3 Clinic for Small Domestic Animals, School of Veterinary Medicine Hannover, Bischofsholer Damm 15, 30173 Hannover, Germany

## Abstract

**Background:**

Studying mechanical properties of canine trabecular bone is important for a better understanding of fracture mechanics or bone disorders and is also needed for numerical simulation of canine femora. No detailed data about elastic moduli and degrees of anisotropy of canine femoral trabecular bone has been published so far, hence the purpose of this study was to measure the elastic modulus of trabecular bone in canine femoral heads by ultrasound testing and to assess whether assuming isotropy of the cancellous bone in femoral heads in dogs is a valid simplification.

**Methods:**

From 8 euthanized dogs, both femora were obtained and cubic specimens were cut from the centre of the femoral head which were oriented along the main pressure and tension trajectories. The specimens were tested using a 100 MHz ultrasound transducer in all three orthogonal directions. The directional elastic moduli of trabecular bone tissue and degrees of anisotropy were calculated.

**Results:**

The elastic modulus along principal bone trajectories was found to be 11.2 GPa ± 0.4, 10.5 ± 2.1 GPa and 10.5 ± 1.8 GPa, respectively. The mean density of the specimens was 1.40 ± 0.09 g/cm^3^. The degrees of anisotropy revealed a significant inverse relationship with specimen densities. No significant differences were found between the elastic moduli in x, y and z directions, suggesting an effective isotropy of trabecular bone tissue in canine femoral heads.

**Discussion:**

This study presents detailed data about elastic moduli of trabecular bone tissue obtained from canine femoral heads. Limitations of the study are the relatively small number of animals investigated and the measurement of whole specimen densities instead of trabecular bone densities which might lead to an underestimation of Young's moduli. Publications on elastic moduli of trabecular bone tissue present results that are similar to our data.

**Conclusion:**

This study provides data about directional elastic moduli and degrees of anisotropy of canine femoral head trabecular bone and might be useful for biomechanical modeling of proximal canine femora.

## Background

The mechanical properties of canine trabecular bone in the femoral head are important for a better understanding of normal biomechanics of the bone and are needed for assessing changes occurring under pathological conditions like osteoarthritis of the hip, osteonecrosis or Legg-Calvé-Perthes disease of the femoral head. Particularly the elastic moduli are important for finite element modeling of the proximal femur. Several methods have been used in the literature for identifying the elastic modulus of bone, such as mechanical testing [1-4], combinations of micro-computed tomography and finite element modeling [4-11], and ultrasonography[12] including acoustic microscopy [13]. Some studies have investigated canine bone [13-19], but to our knowledge no publication has presented any details about directional elastic moduli of canine femoral heads including degrees of anisotropy. Neither is it clear whether assuming isotropy on the tissue level is a justified simplification for trabecular bone in canine femoral heads as Kabel et al.[9] reported in a study of whale bone specimens. Therefore, in this study we determined Young's moduli of trabecular bone obtained from healthy canine femoral heads by ultrasonography. We then calculated degrees of anisotropy and used statistical testing in order to estimate whether assuming isotropy of the trabecular tissue might be a valid simplification.

## Methods

**Figure 1:**
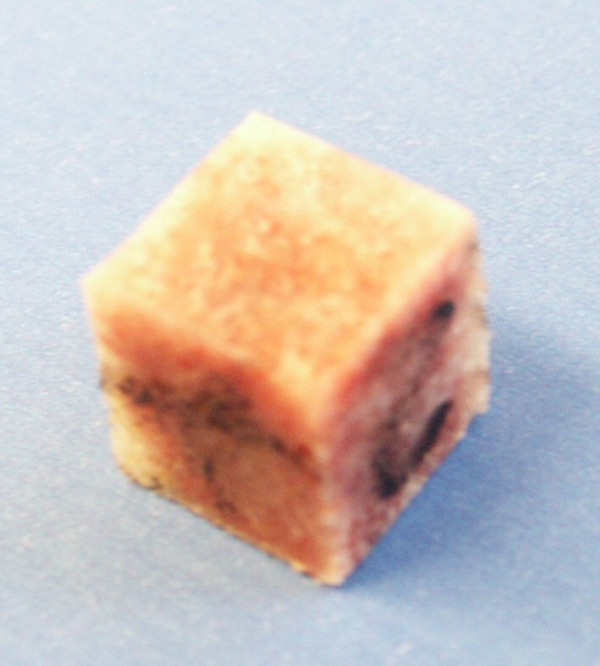
Cubic specimen cut from one canine femoral head.

**Figure 2:**
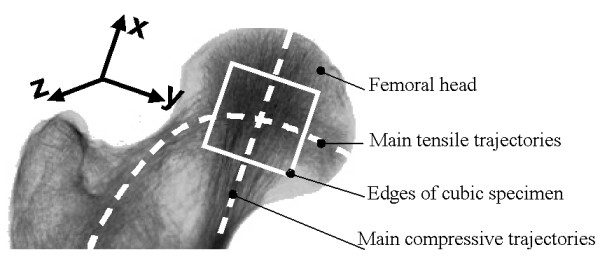
Schematic representation of femoral head and neck. The main tensile and compressive trajectories and the orientation of the cubic specimen and coordinate system are shown.

Eight dogs (weight 30–63 kg) were selected that had been euthanized for several medical reasons. From each dog both femora were obtained and were examined by a veterinarian for signs of metastatic malignant disease, Legg-Calvé-Perthes disease, osteoarthritis of the hip or bone necrosis. An x-ray of the whole femur was obtained with the femoral head and the intertrochanteric region placed directly on the film, and the main pressure and tensile trajectories were marked on the image. The bones were kept moist, wrapped in plastic bags and stored at -21°C. Each bone was placed on the x-ray image and the direction of tension and pressure trajectories were marked on the bone according to the x-ray template. An orthogonal coordinate system was defined (Fig. 2). The positive x-axis was oriented along the main pressure trajectories and the y-axis was aligned with the main tension trajectories. One cubic specimen of 10 × 10 × 10 mm was cut (Fig. 1) from each frozen femoral head using a precision bone saw (Exakt Makro 310 CP, EXAKT Apparatebau, Norderstedt, Germany). The edges of the cubes were cut parallel to the x, y and z axes of the coordinate system. The cubic specimens were weighed using a laboratory scale (Acculab ALC-110.4, Acculab Europe, Göttingen, Germany).

For sonographic testing, a specially designed device with a custom-made ultrasound transducer (Institute of Materials Science, University of Hannover) was used. An ultrasound frequency of over 2 MHz was chosen for measuring the material properties of trabecular bone tissue[20]. The ultrasound frequency was adjusted so that a clear signal could be detected by the ultrasound receiver. A frequency of 100 MHz was chosen because ultrasound signals remained undetectable when using lower frequencies even with maximum power. The cubic specimens were placed in a container after thawing and immersed in standard Ringer's solution at room temperature. An ultrasound receiver was placed at the surface of the cube opposite the transducer which was also directly touching the specimen surface, and the runtime through the bone material of each bone cube (n = 16) was recorded ten times in all three orthogonal directions. The edge lengths of each cube were measured using the digital image analysis system IMAGE C^® ^(IMTRONIC GmbH, Berlin, Germany), and the specimen volumes were calculated. Specimen densities were determined by equation (1):



where ρ is specimen density, m is specimen mass and V is the specimen volume

The ultrasound wave runtimes were processed by excluding the minimum and maximum results of the ten subsequent measurements, and the average of the remaining results was calculated. The transmission velocity was calculated by equation (2) and the elastic modulus was determined using equation (3).



where c_long _is the transmission velocity, s denotes the edge length of the specimen and equals the distance between ultrasound transmitter and receiver which are placed in direct contact with the opposing specimen surfaces, t_1 _and t_0 _the time at reception and sending of the ultrasound wave, respectively.



where E_x,y,z _is Young's modulus along x, y and z axes and ρ is the specimen density calculated from equation (1). C_long _is the ultrasound velocity calculated from specimen edge length and transmission time (see equation (2))

Mean density and mean Young's moduli of the specimens and standard deviations were determined using the statistical software package SPSS 12.0 (SPSS, Chicago, USA). Strong correlations between density and elastic moduli could be expected from equation (3), nonetheless Pearson correlations were calculated for confirmation. The directional elastic moduli were checked for significant differences using one-way ANOVA (analysis of variance). The degrees of anisotropy [4,21](E_x_/E_z; _E_x_/E_y_; E_z_/E_y_) were calculated and subsequently checked for a significant relationship with specimen density using Pearson correlations.

## Results

**Figure 3:**
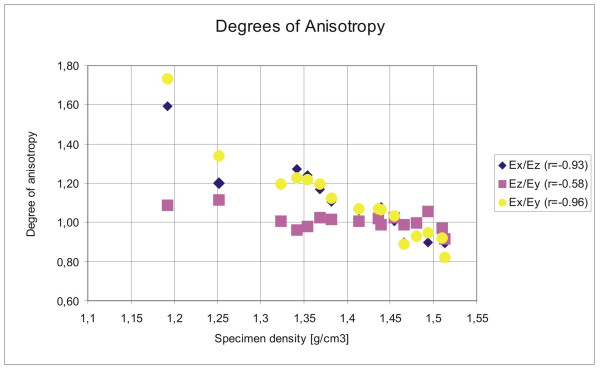
Degrees of anisotropy in dependence of specimen density. Correlation coefficients are listed in the symbol legend.

The edge lengths of the bone specimens varied by ± 1 % (± 0.1 mm). Elastic moduli in the bone specimens ranging from 6.3 to 14.3 GPa were found. The elastic moduli in X, Y and Z direction were 11.2 ± 0.4 GPa, 10.5 ± 2.1 GPa and 10.5 ± 1.8 GPa, respectively. Minimum, maximum, mean values and standard deviations of bone sample density and directional Young's moduli are listed in Table 1. Pearson correlations between density and directional Young's moduli (E_x_, E_y _and E_z_) were significant as could be expected from equation (3) (p < 0.005). The degrees of anisotropy ranged from 0.82 to 1.59 and were significantly correlated with specimen density (Table 2, Figure 3).

**Table 1:** Minimum, maximum and mean values for sample densities and directional Young's moduli Moduli are arranged according to testing direction (along x, y and z axes, see fig. 2).

	**Minimum**	**Maximum**	**Mean**	**Standard deviation**
**Density [g/cm^3^]**	1.19	1.51	1.40	0.09
**E_X _[MPa]**	10600	11760	11217	376
**E_Y _[MPa]**	6283	14285	10459	2071
**E_Z _[MPa]**	6832	13097	10506	1839

**Table 2:** Degrees of anisotropy The minimum and maximum degrees of anisotropy (range), the mean values, standard deviations (SD) and the correlation coefficients (r) indicating correlation of the respective degree of anisotropy with specimen density ρ. p designates the significance probability (p-value).

	**Range**	**Mean**	**SD**	**r**	**p**
**E_x_/E_z_**	0.89–1.59	1.10	0.18	-0,93	0.000
**E_z_/E_y_**	0.92–1.11	1.01	0.05	-0,58	0.009
**E_x_/E_y_**	0.82–1.73	1.11	0.22	-0,96	0.000

## Discussion

This study presents detailed data about mechanical properties of canine femoral trabecular bone tissue and degrees of anisotropy. Despite the strengths of our work, some limitations have to be noted.

The correct calculation of the sample volume depends on exactly cubic specimens, but deviations might occur due to errors in the sawing technique. We found a maximum variation of edge length of ± 0.1 mm (1%) in our specimens so this error appears to be negligible.

The apparent densities of the specimens were calculated by weighing whole samples and measuring sample volumes instead of calculating the densities of ashed samples[2,19] or cleaning the bone marrow out of the specimens using water jets prior to measurement. According to Rho [20] ultrasonic waves at frequencies of >2 MHz travel along the trabecular material and allow calculation of the elasticity of the trabecular bone material rather than the elasticity of whole specimens which is investigated by conventional compression testing. Considering this statement, in our study using a 100 MHz ultrasound transducer the application of apparent densities rather than the densities of the trabecular bone material might lead to an conspicuous underestimation of the elastic modulus (equation (3)) because it is expected that the density of the trabecular bone tissue is higher than the density of whole specimens. However, Kang et al. [22] measured densities of cylindrical trabecular bone specimens from canine femoral heads and reported a mean density of 1.17 ± 17 g/cm^3 ^for whole bone cylinders after cleaning and 0.65 ± 0.09 g/cm^3 ^for ashed samples which is much lower than our results. The lower densities might be caused by the specimen volume that was used by the authors: they geometrically measured the volume of the cylindrical bone specimens so that a considerable intertrabecular volume is included and bone tissue densities are underestimated according to equation (1). Hence the results of Kang's publication can not be used for comparison or correction of our density data.

It is noticeable that in our study we found a small relative standard deviation (SD/mean) in apparent densities and Young's modulus in x direction (6.6 % and 3.3 %) although we used femora from a heterogeneous selection of different breeds. However, the relative SD in y and z directions was computed to be 19.5 % and 17.5 %, respectively. It is not clear whether the broader range of elastic moduli in these directions reflects real differences or whether it might be caused by a less accurate positioning of the saw when cutting the specimens from the femoral heads.

The significant relationship between the specimen density ρ and directional elastic moduli found in our study was to be expected because Young's modulus was calculated from ρ. This significant relationship was also described in a study using compression testing of bone cubes from human donors (0.74 <= r <= 0.84; p < 0.001) [2].

No significant differences were found between directional elastic moduli (p = 0.34). This result could support the concept of an "effective" isotropic elastic tissue modulus as described by Kabel et al.[9].

Several works have been published concerning elastic moduli of canine or human bone. Studies that investigated the apparent elastic modulus of human bone specimens using mechanical testing and finite element models found much lower elastic moduli[4,21] than we did in our work. Kang et al. [22] reported elastic moduli of trabecular specimens from canine femoral heads of 428 ± 237 MPa which is also much lower than what we observed in our study; their results were obtained by conventional compression testing. Several other studies have been carried out investigating elastic moduli of canine trabecular bone specimens[14,16,19]. Those results are not comparable with our data because the authors measured elastic moduli of whole specimens rather than Young's moduli on the tissue level. Additionally, Odgaard et al. reported that conventional compression testing underestimates Young's modulus by about 20%[23]. Keaveny et al. [24] found a percentage difference in modulus when using platens compression testing of up to 86%. They recommend using the endcap technique for obtaining more accurate data, which however restricts testing to one direction. Jacobs et al. [25] investigated porous samples made from bone cement. Using finite element modeling and mechanical testing, they found that the mean error when using parallel platen compression testing was 8 or 15% depending on FE mesh size and was reduced to 2 or 0.5% with the endcap technique.

Only one study investigating elastic moduli of canine trabecular bone tissue is available to our knowledge: Jorgensen and Kundu [13] used a 1 GHz acoustic microscope for examining a trabecular strut obtained from a canine distal femur. They computed a mean Young's modulus of 19.9 ± 2.5 GPa which is higher than our results; this could be caused by a different trabecular structure and higher bone volume fraction in the distal femur. The authors state that anisotropy was clearly detected at micrometer level, but no further quantification is given.

More studies are available reporting mechanical properties of human trabecular bone. Rho [20] found an elastic modulus of 14.9 ± 1.7 GPa in trabecular bone specimens from human tibiae using a 2.25 MHz transducer. This modulus is higher than in our data but within the same order of magnitude. Ashman and Rho [26] measured elastic moduli from three human trabecular bone specimens using an ultrasonic technique and found a mean elastic modulus of 13.0 GPa which is about 30% higher than in our study of canine bone. This observation is supported by Kuhn's [19] assertion that elastic moduli are higher in human trabecular bone than in canine bone. Our results are further supported by Zysset et al. [27] who used a nanoindentation technique and found average elastic moduli in trabecular lamellae of human femoral necks of 11.4 ± 5.6 GPa.

## Conclusion

Our study provides detailed data about elastic moduli and degrees of anisotropy of canine femoral bone tissue. No significant differences between directional Young's moduli were found indicating that the concept of an effective isotropy of trabecular bone in canine femoral heads might be a justified simplification. The degrees of anisotropy were highly correlated with specimen densities. The results of elastic moduli are comparable to similar studies of canine and human trabecular bone tissue [13,20,26,27].

## Authors' contributions

TP carried out the statistical analysis of the results, took part in the detailed design of the study and prepared the manuscript. AB designed the study, prepared the bone specimens, conducted the ultrasound measurements and calculated the elastic moduli. UV and AML obtained the canine femora, examined the bone for exclusion criteria and prepared the bone specimens. BAB, IN and HW designed the study concept from a technical and medical perspective, respectively, and corrected the manuscript.
