# Correction: Mechanical properties of femoral trabecular bone in dogs

**DOI:** 10.1186/1475-925X-4-66

**Published:** 2005-12-07

**Authors:** Thomas Pressel, Anas Bouguecha, Ute Vogt, Andrea Meyer-Lindenberg, Bernd-Arno Behrens, Ingo Nolte, Henning Windhagen

**Affiliations:** 1 Department of Orthopaedic Surgery, Hannover Medical School, Anna-von-Borries-Str. 1–7, 30625 Hannover, Germany; 2 Institute of Metal Forming and Metal Forming Machine Tools, University of Hannover, Schönebecker Allee 2, 30823 Garbsen, Germany; 3 Clinic for Small Domestic Animals, School of Veterinary Medicine Hannover, Bischofsholer Damm 15, 30173 Hannover, Germany

After the publication of this work [1], we became aware of the fact that the frequency of the ultrasound transmitter that we used for determining the elastic moduli of the trabecular bone specimens was not correctly specified. The oscillation frequency of the ultrasound transmitter was 2 MHz (and not 100 MHz as stated in our work) while we used a sampling rate of 100 MHz. In our publication, the oscillation frequency and sampling rate were confounded. Therefore also the statement in the discussion that we might have determined elastic moduli of trabecular bone tissue rather than the elastic properties of whole specimens because we used an ultrasound frequency > 2 MHz is wrong and has to be omitted.

For measurement, the cubic bone specimens were not immersed in Ringer's solution but only were kept moist all the time.

Apart from these corrections concerning the methods and interpretation of the data, the results reported in our publication and the conclusions are absolutely correct.

We apologize for the inconvenience that this inaccuracy may have caused.
